# First-in-Human Evaluation of Site-Specifically Labeled ^89^Zr-Pertuzumab in Patients with HER2-Positive Breast Cancer

**DOI:** 10.2967/jnumed.123.266392

**Published:** 2024-03

**Authors:** Randy Yeh, Joseph A. O’Donoghue, Vetri Sudar Jayaprakasam, Audrey Mauguen, Ryan Min, Sue Park, Julia P. Brockway, Jacqueline F. Bromberg, W Iris Zhi, Mark E. Robson, Rachel Sanford, Shanu Modi, Brian J. Agnew, Serge K. Lyashchenko, Jason S. Lewis, Gary A. Ulaner, Brian M. Zeglis

**Affiliations:** 1 Department of Radiology, Memorial Sloan Kettering Cancer Center, New York, New York;; 2 Department of Radiology, Weill Cornell Medical College, New York, New York;; 3 Department of Medical Physics, Memorial Sloan Kettering Cancer Center, New York, New York;; 4 Department of Biostatistics and Epidemiology, Memorial Sloan Kettering Cancer Center, New York, New York;; 5 Department of Medicine, Memorial Sloan Kettering Cancer Center, New York, New York;; 6 Department of Medicine, Weill Cornell Medical College, New York, New York;; 7 Biosciences Division, Thermo Fisher Scientific, Eugene, Oregon;; 8 Program in Molecular Pharmacology, Memorial Sloan Kettering Cancer Center, New York, New York;; 9 Molecular Imaging and Therapy, Hoag Family Cancer Institute, Newport Beach, California;; 10 Departments of Radiology and Translational Genomics, University of Southern California, Los Angeles, California; and; 11 Department of Chemistry, Hunter College, New York, New York

**Keywords:** breast cancer, HER2, immuno-PET, radioimmunoconjugates

## Abstract

Radioimmunoconjugates targeting human epidermal growth factor receptor 2 (HER2) have shown potential to noninvasively visualize HER2-positive tumors. However, the stochastic approach that has been traditionally used to radiolabel these antibodies yields poorly defined and heterogeneous products with suboptimal in vivo performance. Here, we describe a first-in-human PET study on patients with HER2-positive breast cancer evaluating the safety, biodistribution, and dosimetry of ^89^Zr-site-specific (ss)-pertuzumab PET, a site-specifically labeled radioimmunoconjugate designed to circumvent the limitations of random stochastic lysine labeling. **Methods:** Six patients with HER2-positive metastatic breast cancer were enrolled in a prospective clinical trial. Pertuzumab was site-specifically modified with desferrioxamine (DFO) via a novel chemoenzymatic strategy and subsequently labeled with ^89^Zr. Patients were administered 74 MBq of ^89^Zr-ss-pertuzumab in 20 mg of total antibody intravenously and underwent PET/CT at 1 d, 3–4 d, and 5–8 d after injection. PET imaging, whole-body probe counts, and blood draws were performed to assess the pharmacokinetics, biodistribution, and dosimetry. **Results:** ^89^Zr-ss-pertuzumab PET/CT was used to assess HER2 status and heterogeneity to guide biopsy and decide the next line of treatment at progression. The radioimmunoconjugate was able to detect known sites of malignancy, suggesting that these tumor lesions were HER2-positive. The optimal imaging time point was 5–8 d after administration, and no toxicities were observed. Dosimetry estimates from OLINDA showed that the organs receiving the highest doses (mean ± SD) were kidney (1.8 ± 0.5 mGy/MBq), liver (1.7 ± 0.3 mGy/MBq), and heart wall (1.2 ± 0.1 mGy/MBq). The average effective dose for ^89^Zr-ss-pertuzumab was 0.54 ± 0.03 mSv/MBq, which was comparable to both stochastically lysine-labeled ^89^Zr-DFO-pertuzumab and ^89^Zr-DFO-trastuzumab. One patient underwent PET/CT with both ^89^Zr-ss-pertuzumab and ^89^Zr-DFO-pertuzumab 1 mo apart, with ^89^Zr-ss-pertuzumab demonstrating improved lesion detection and higher tracer avidity. **Conclusion:** This study demonstrated the safety, dosimetry, and potential clinical applications of ^89^Zr-ss-pertuzumab PET/CT. ^89^Zr-ss-pertuzumab may detect more lesions than ^89^Zr-DFO-pertuzumab. Potential clinical applications include real-time evaluation of HER2 status to guide biopsy and assist in treatment decisions.

## Visual Abstract

**Figure:**
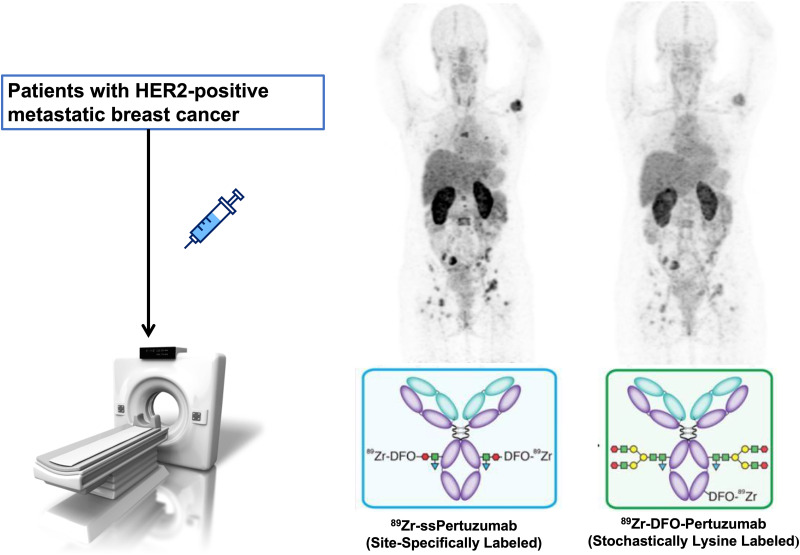

Over the past 2 decades, human epidermal growth factor receptor 2 (HER2) has emerged as a critical prognostic biomarker in patients with metastatic breast cancer (mBC), as well as a vital target for therapeutics such as trastuzumab and pertuzumab. HER2 status is traditionally determined via immunohistochemistry (IHC) for protein expression or in situ hybridization for gene amplification (1). Determining HER2 status via single-site biopsies is indeed useful and remains the gold standard for qualifying patients for HER2-targeted therapies. However, this approach inevitably fails to capture intertumoral HER2 expression heterogeneity, and biopsies from multiple lesions are not feasible or routinely performed in clinical practice. In fact, HER2 discordance between primary and metastatic tumors can be as high as 25% (2), and the limitations of single-site biopsies for heterogeneity may in part explain the mixed or discordant responses in some patients with HER2-positive mBC to HER2-targeted therapies.

To address these challenges, PET imaging with radiolabeled HER2-targeted antibodies was developed as a noninvasive approach to evaluate HER2 expression in both primary tumors and metastatic lesions, thereby providing a snapshot of receptor heterogeneity throughout the body. In 2010, Dijkers et al. from the University of Groningen introduced HER2 PET by evaluating ^89^Zr-trastuzumab PET in mBC patients (3). This group’s pioneering work continued in subsequent studies, including investigating ^89^Zr-trastuzumab to assess HER2 heterogeneity and predict response to trastuzumab emtansine in the ZEPHIR trial (4) and to assist in clinical decision-making when HER2 status could not be determined (5). More recently, Dehdashti et al. showed that ^89^Zr-trastuzumab PET could discriminate between HER2-positive and HER2-negative lesions in mBC patients (6). Our group has performed several clinical trials to evaluate HER2-targeted PET radiotracers in both breast and gastroesophageal cancers (7–12), beginning with ^89^Zr-desferrioxamine (DFO)-trastuzumab (7–9,12) and then ^89^Zr-DFO-pertuzumab (10,11). These studies demonstrated the clinical potential of HER2 PET for detecting HER2-positive tumor lesions, evaluating HER2 heterogeneity, and identifying occult HER2-positive lesions in patients with HER2-negative breast cancer (7–12).

Several weaknesses of ^89^Zr-labeled HER2-targeting PET tracers have been elucidated over the last decade, including absent or low uptake in HER2-positive metastases (false negatives), as well as high background uptake in the liver and bone marrow that reduces sensitivity for detecting HER2-positive lesions. In addition, a high rate of false positives for HER2-positive tumor was observed in patients who subsequently underwent biopsy of tracer-avid lesions (7,8,11).

One possible route to improving the performance of ^89^Zr-labeled HER2-targeting PET tracers is site-specific (ss) modification. In general, radioimmunoconjugates are created by randomly attaching chelators, for example DFO, to lysines within the antibody and then labeling the resultant immunoconjugate with the radiometal of choice, for example ^89^Zr. However, because antibodies have upward of 40 lysines distributed through their macromolecular structure, this approach—although undeniably facile—inevitably produces poorly defined and complex heterogeneous mixtures of regioisomers that can exhibit suboptimal in vitro and in vivo behavior, including attenuated immunoreactivity and increased uptake in nontarget tissues (13,14). Although pertuzumab does contain lysine residues within its complementarity-determining regions (15), the antibody was chosen for this investigation because a stochastically labeled variant—^89^Zr-DFO-pertuzumab—has already been clinically translated and thus could provide a point of reference for the clinical performance of the site-specifically modified radioimmunoconjugate.

Over the last several years, we have developed and validated a novel chemoenzymatic method for the synthesis of well-defined and homogeneous radioimmunoconjugates (14,16,17). This strategy relies on a pair of enzymes—EndoS and GalT(Y289L)—to incorporate azide-containing sugars into the heavy-chain glycans of the antibody’s Fc region and then leverages the strain-promoted azide-alkyne cycloaddition click reaction to attach dibenzocyclooctyne-bearing chelators to these artificial sugars (14). Using this methodology, we have previously synthesized ^89^Zr-ss-pertuzumab in high yield and high specific activity and demonstrated its homogeneity, immunoreactivity, and stability. Furthermore, in preclinical models of HER2-positive breast cancer, ^89^Zr-ss-pertuzumab exhibited excellent in vivo behavior, in several cases surpassing that of its traditionally synthesized progenitor, stochastically lysine labeled ^89^Zr-DFO-pertuzumab (16). Yet despite these clear advantages, a site-specifically modified radioimmunoconjugate has never, to the best of our knowledge, been translated to the clinic. Here, we present the results of a first-in-human clinical trial of ^89^Zr-ss-pertuzumab in HER2-positive mBC patients focused on the safety, pharmacokinetic profile, dosimetry, and potential clinical applications of this site-specifically modified radioimmunoconjugate.

## MATERIALS AND METHODS

### Study Design

This study was a prospective, single-center, single-arm, and open-label imaging trial. The study protocol was approved by the Memorial Sloan Kettering Cancer Institutional Review Board and was registered with the National Library of Medicine (ClinicalTrials.gov identifier NCT04692831). All patients gave written informed consent.

### Patients

Patients with HER2-positive mBC were identified and recruited from the breast medical oncology clinics. HER2 status was defined according to American Society of Clinical Oncology/College of American Pathologist guidelines (1), with HER2-positive defined as an HER2 IHC score of 3+ or an IHC score of 2+ with HER2 amplification on fluorescence in situ hybridization (FISH) as defined by a HER2–to–CEP17 ratio of at least 2.0. Tumor samples with an IHC score of 0, 1+, or 2+ and FISH-negative were defined as HER2-negative. Inclusion criteria were adult patients (>18 y old) with biopsy-proven HER2-positive primary malignancy or metastatic disease; biopsy-proven metastatic disease; at least 5 malignant lesions on CT, MRI, or ^18^F-FDG PET/CT within 60 d of the protocol; and an Eastern Cooperative Oncology Group performance score of 0–2 (18). Exclusion criteria were creatinine more than 2 times the normal limit, aspartate transaminase or alkaline phosphatase more than 2 times the normal limit within 8 wk, life expectancy of less than 3 mo, pregnancy or lactation, and patients who could not undergo PET/CT.

### Preparation of ^89^Zr-ss-Pertuzumab

^89^Zr-ss-pertuzumab was manufactured by the Memorial Sloan Kettering Radiochemistry and Molecular Imaging Probes Core Facility in compliance with a U.S. Food and Drug Administration Investigational New Drug application (investigational new drug 153644). Clinical-grade pertuzumab (Perjeta; Genentech) was site-specifically modified with DFO and then radiolabeled with ^89^Zr, a positron-emitting radiometal with a 78.4-h radioactive half-life. The conjugation was performed using a previously described methodology (12) in which a pair of enzymes, EndoS and GalT(Y289L) (Thermo Fisher Scientific), was used in conjunction with bioorthogonal click chemistry to append DFO to the heavy-chain glycans of the Fc region’s CH2 domain (14). The final drug product, ^89^Zr-ss-pertuzumab, underwent quality control testing before batch release for patient administration, to ensure conformance with specifications for radiochemical purity, radioimmunoreactivity, endotoxin content, sterilizing filter integrity, pH, visual appearance, radionuclidic identity verification, and sterility testing.

### Administration of ^89^Zr-ss-Pertuzumab

All patients received 74 MBq ± 10% of ^89^Zr-ss-pertuzumab intravenously in 20 mg of total antibody mass with an approximate mass ratio of 18 mg of cold, nonradiolabeled antibody and 2 mg of ^89^Zr-labeled antibody. After placement of an intravenous line, nonradiolabeled and unmodified pertuzumab was administered over 5 min to help reduce nonspecific uptake as previously described for HER2-targeted PET tracers (3,7). Then, ^89^Zr-ss-pertuzumab was administered as an intravenous push. The line was primed and subsequently flushed with 5% human serum albumin solution. Patients were monitored for 2 h after administration and subsequently with a follow-up phone call by a study physician 1–3 d later. Any adverse effects were graded using the Common Terminology Criteria for Adverse Events (version 4).

### ^89^Zr-ss-Pertuzumab PET/CT Imaging

All patients returned for serial PET/CT imaging at 1, 3–4, and 5–8 d after administration. Patients underwent PET/CT from the top of the skull to the mid thigh on a dedicated research PET/CT scanner (Discovery PET/CT 710; GE Healthcare), with low-dose CT (80 mA) for attenuation correction and lesion localization. PET imaging was performed at 6–7 bed positions with a total imaging time of no more than 1 h (8 min/bed position). Images were reconstructed using our standard method with 3-dimensional ordered-subsets expectation maximization with 2 iterations, 16 subsets, and a postreconstruction gaussian filter of 7 mm, as well as Q.clear reconstruction (GE Healthcare).

^89^Zr-ss-pertuzumab PET/CT images were interpreted by a nuclear radiologist experienced in HER2-targeted PET. The interpreting radiologist knew the patient’s clinical history and prior imaging results. Physiologic uptake of ^89^Zr-ss-pertuzumab was expected in the blood pool, liver, spleen, and kidneys on the basis of our experience with ^89^Zr-DFO-pertuzumab (10). Foci of radiotracer uptake in nonphysiologic areas and greater than adjacent background were considered positive for HER2-positive tumor. Three-dimensional volumes of interest were drawn on the PET/CT images using a dedicated workstation (Hermes Medical Solutions) over the cardiac blood pool, aortic arch, and normal liver, kidney, spleen, and lung. In addition, for patients with discernible uptake in the gastrointestinal tract or lesions, corresponding volumes of interest were generated for the whole gastrointestinal tract and up to 3 index lesions using a thresholding approach. SUVs normalized to lean body mass were quantified.

### Whole-Body and Serum Clearance Measurements

Whole-body clearance was determined by serial measurements of count rate using a 12.7-cm-thick NaI(Tl) scintillation detector at a fixed distance of 3 m from the patient. Background-corrected geometric mean counts were obtained before and after the first voiding and subsequently at the times of the PET scans. Count rates were normalized to the immediate postinfusion value (taken as 100%) to yield relative retained activities (as a percentage).

Serial blood samples were obtained before radiotracer administration and at approximately 30 min, 60 min, and 2 h after administration and on subsequent days of each PET scan (*n* = 7 samples total). Counts in aliquots of serum were obtained using a well-type detector (Wallac Wizard 1480 γ-counter; Perkin Elmer) and expressed as percentage injected activity per liter.

Monoexponential functions were fitted to the whole-body probe data, and biexponential functions were fitted to the serum activity concentration data, using SAAM software (19). Time-integrated activity coefficients (TIACs) for the whole body and for serum were determined from these data. Serum data were also used to determine pharmacokinetic parameters, including concentration at time zero, the distribution volume of the central compartment, area under the curve (AUC), and systemic clearance. The total percentage injected activity initially present in the serum was estimated by multiplying the percentage injected activity per liter in serum at time zero by the patient’s estimated plasma volume determined from a nomogram (20).

### Normal-Tissue Dosimetry

Normal-tissue dose estimates were derived as described previously (9,21). In brief, the AUCs of image-derived activity concentration per unit mass (kBq/g) were estimated by trapezoidal integration. Whole-organ AUCs were estimated by multiplying the activity concentration AUCs by the projected organ mass. TIACs were derived by dividing whole-organ AUCs by the administered activity. Corresponding values for heart contents and red marrow were estimated from the serum TIAC (22). TIACs for the remainder of the body were derived by subtracting all individually estimated TIACs from the whole-body TIAC. Thereafter, absorbed radiation doses to individual organs were estimated using the OLINDA/EXM software application (23). Normal-tissue dosimetry estimates for ^89^Zr-ss-pertuzumab were compared with published values for ^89^Zr-DFO-pertuzumab and ^89^Zr-DFO-trastuzumab.

### Statistics

Kinetic parameters and absorbed dose estimates were calculated for each patient and summarized using descriptive statistics including median or mean and SD.

## RESULTS

### Patient Characteristics

Between May 2021 and May 2022, 6 patients (5 female, 1 male; median age, 51 y; range, 41–69) with HER2-positive mBC completed the study protocol. All patients underwent serial imaging on days 1, 3–5, and 6–8 after injection of ^89^Zr-ss-pertuzumab. Table 1 summarizes the patient characteristics.

**TABLE 1.** Patient Characteristics

Patient no.	Age (y)	Sex	Avidity		Concordance
			^18^F-FDG	^89^Zr-ss-pertuzumab	
1	50	F	Nodal, hepatic, right lung	Nodal, hepatic, right lung	100%
2	48	F	Osseous	Osseous	100%
3	41	F	Right breast, nodal, hepatic, osseous	None (interval treatment response)	0%
4	57	M	Hepatic, osseous	Osseous	7.4%
5	52	F	Osseous, lung	Solitary osseous (sternum)	20%
6	69	F	Osseous, nodal	Osseous	80%

### Detection of Sites of Known Malignancy with *^8^*^9^Zr-ss-Pertuzumab PET

All patients had sites of malignancy determined on ^18^F-FDG PET/CT performed within 60 d of study enrollment. Known osseous metastatic disease was present in 5 patients, hepatic disease in 3 patients, nodal disease in 3 patients, lung disease in 2 patients, and primary breast malignancy in 1 patient. ^89^Zr-ss-pertuzumab PET scans were positive in 5 patients and negative in 1 patient. Of 5 patients with positive scans, complete concordance between ^18^F-FDG- and ^89^Zr-ss-pertuzumab–avid lesions at sites of known malignancy was observed in 2 patients. The 3 other patients demonstrated HER2 intertumoral heterogeneity, with 5% concordance, 20% concordance, and 80% concordance in patients 4, 5, and 6, respectively. Sites of known malignancy and ^89^Zr-ss-pertuzumab PET detection are summarized in Table 1.

### Adverse Events

All 6 patients underwent intravenous administration of ^89^Zr-ss-pertuzumab. No related side effects were observed or reported. The mean (±SD) of the administered mass of pertuzumab was 19.1 ± 0.375 mg (range, 18.5–19.5 mg). The mean administered activity was 73.1 MBq (range, 70.7–74.4 MBq). There were no adverse or clinically detectable pharmacologic effects in any of the 6 subjects. No significant changes in vital signs were observed.

### Pharmacokinetics

**FIGURE 1.**
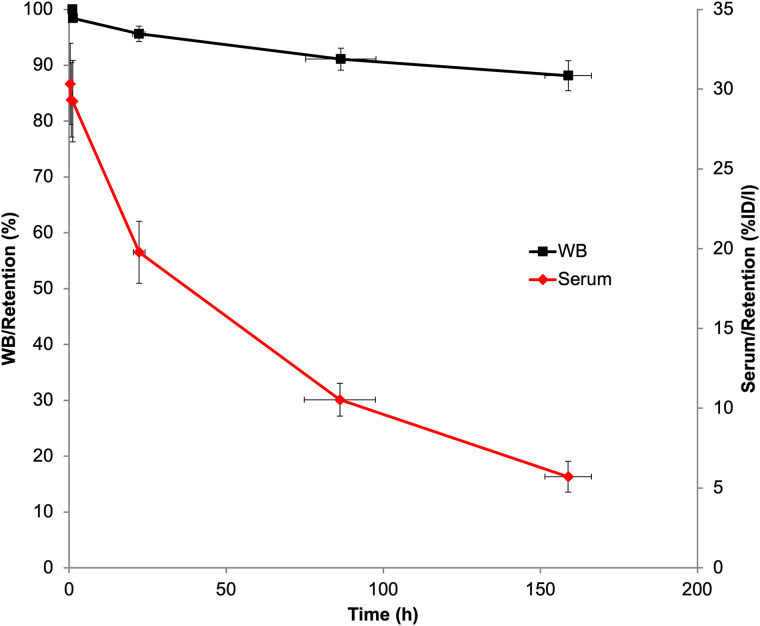
Summed whole-body and serum biologic clearance curves for ^89^Zr-ss-pertuzumab in 6 patients. Error bars indicate SE of mean. %ID/l = percentage injected dose per liter; WB = whole body.

Whole-body and serum clearance conformed to mono- and biexponential kinetics, respectively. Summed biologic clearance data are shown in Figure 1, and summary statistics for the clearance parameters are provided in Supplemental Table 1 (supplemental materials are available at http://jnm.snmjournals.org).

### Biodistribution and Normal-Tissue Dose Estimates

Similar to other HER2-targeted PET radiotracers, the optimal imaging time point for tumor visualization was 5–8 d after administration, when the best contrast was observed between tumor foci and background uptake. The early images at 1 d after administration showed primarily blood pool, with little to no observable uptake in tumor lesions. Lesion uptake gradually increased with time and was highest at the last time point. Uptake in all normal tissues except kidney decreased with time. Kidney uptake continued to increase over the period of observation.

Absorbed dose estimates for normal tissues are summarized in Supplemental Table 2. The organs receiving the highest mean doses were kidney (1.8 ± 0.5 mGy/MBq), liver (1.7 ± 0.3 mGy/MBq), heart wall (1.2 ± 0.1 mGy/MBq), and lung (1.2 ± 0.2 mGy/MBq).

### Comparison of ^89^Zr-ss-Pertuzumab Dosimetry with ^89^Zr-DFO-Pertuzumab and ^89^Zr-Trastuzumab Dosimetry

**FIGURE 2.**
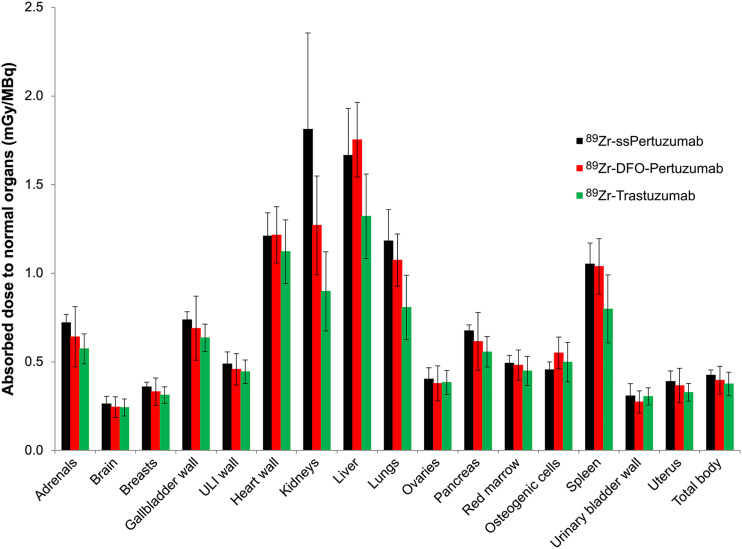
Comparison of radiation dosimetry for HER2 immuno-PET radiotracers. ULI = upper large intestinal.

The average effective dose was 0.54 ± 0.03 mSv/MBq, which is the same as that reported for ^89^Zr-DFO-pertuzumab (0.54 ± 0.07 mSv/MBq; *P* > 0.05) (10) but higher than that reported for ^89^Zr-trastuzumab (0.48 ± 0.06 mSv/MBq; *P* < 0.05) (9). The comparatively lower absorbed doses reported for ^89^Zr-trastuzumab are likely related to the large fraction of male vs. female patients (8 vs. 2) in the original esophagogastric cancer study. Figure 2 compares the biodistribution and absorbed dose estimates for the 3 HER2-targeting ^89^Zr-immuno-PET probes.

### Tumor Imaging with ^89^Zr-ss-Pertuzumab PET/CT

**FIGURE 3.**
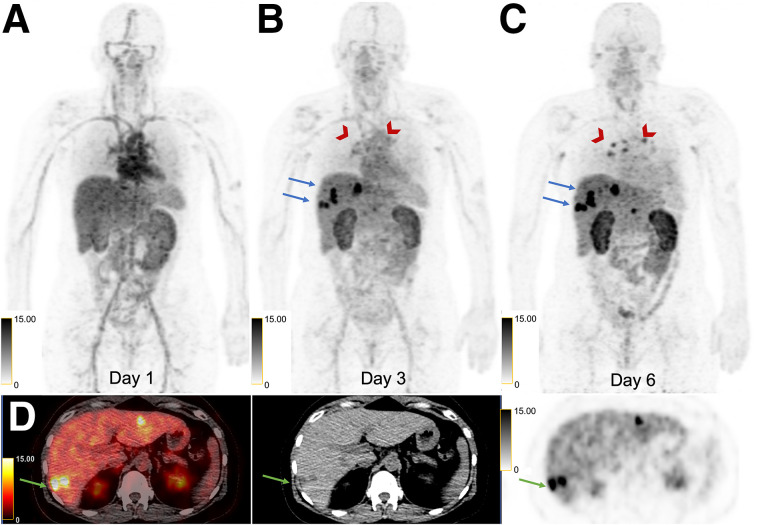
50-y-old woman with HER2-positive mBC and known right lung, thoracic nodal, and liver metastases. (A–C) Sequential maximum-intensity projection PET images at 1 d (A), 3 d (B), and 6 d (C) after administration of ^89^Zr-ss-pertuzumab demonstrating gradually increasing uptake in thoracic nodes (arrowheads) and hepatic lesions (arrows) over time. Decreasing blood pool and slightly decreasing liver background uptake was observed on serial imaging, with increasing kidney uptake. (D) Axial ^89^Zr-ss-pertuzumab PET/CT, CT, and PET images at 6 d demonstrating a few avid hepatic lesions, of which segment 6 lesion (arrows) was selected for biopsy.

Patient 1 had an estrogen receptor (ER)–positive, progesterone receptor (PR)–positive, HER2-positive (IHC score, 2+; FISH ratio, 2.0) right-breast invasive ductal carcinoma (IDC) diagnosed in 2016 and underwent mastectomy followed by adjuvant therapy. In November 2020, she developed metastatic disease to the thoracic nodes and lungs. A subcarinal node was biopsied twice and found to be HER2-negative (first biopsy: IHC score, 0; second biopsy: IHC score, 2+; FISH ratio, 1.2). She started taking anastrozole and palbociclib but subsequently developed disease progression with new hepatic metastases. Given the discordant HER2 results between the breast primary and the metastatic disease, she was referred for ^89^Zr-ss-pertuzumab PET/CT to help select a liver lesion for biopsy to maximize the chances of HER2-positive disease and subsequent HER2-targeted therapy. She had thoracic nodes, liver lesions, and a right lung nodule avid for ^18^F-FDG at enrollment. There was gradually increasing ^89^Zr-ss-pertuzumab avidity of all lesions on days 3 and 6, with no avidity above the background level on day 1 (Fig. 3). Decreasing background blood pool and slightly decreasing liver uptake from days 1 to 6 allowed for optimal visualization of lesions on day 6. The liver lesions were the most avid, with a segment 4A/8 lesion demonstrating an SUV_max_ of 11.4 (equal to liver background), 20.7, and 35.0, on days 1, 3, and 6, respectively. The second most avid liver lesion, in segment 6 (SUV_max_, 30.0), was selected for biopsy given the ease of accessibility and high uptake. However, the biopsy found the lesion to be HER2-negative (IHC score, 1+).

Patient 2 had an ER-positive, PR-positive, HER2-positive (IHC score, 1–2+; FISH ratio, 3.0) right-breast IDC diagnosed in 2018 and underwent lumpectomy. The patient declined adjuvant therapy and developed right-breast, axilla, and bone recurrence in 2019, with HER2-negative disease (IHC score, 1+) in both breast and bone. The patient received paclitaxel, trastuzumab, and pertuzumab followed by maintenance trastuzumab and pertuzumab until progression of bone metastasis in 2021. She then received trastuzumab emtansine for a few months with further bony progression. Given the HER2 discordance, a repeat bone biopsy was planned, and the patient was referred for ^89^Zr-ss-pertuzumab PET/CT to help select a bone lesion. She had numerous ^18^F-FDG–avid osseous lesions at enrollment, all with increasing ^89^Zr-ss-pertuzumab avidity on days 1, 5, and 7. A right sacral lesion was the most avid (SUV_max_, 6.5, 20.7, and 22.3 on days 1, 5, and 7, respectively) and was selected for biopsy. The sclerotic and most conspicuous component of this right sacral lesion had the least tracer avidity, and ^89^Zr-ss-pertuzumab PET/CT directed the CT-guided biopsy toward an adjacent more avid component without a CT correlate. However, biopsy showed HER2 negativity (IHC score, 1–2+; FISH ratio, 1.3), and as a result, the patient received palbociclib, letrozole, and leuprorelin instead of trastuzumab deruxtecan (T-DXd).

**FIGURE 4.**
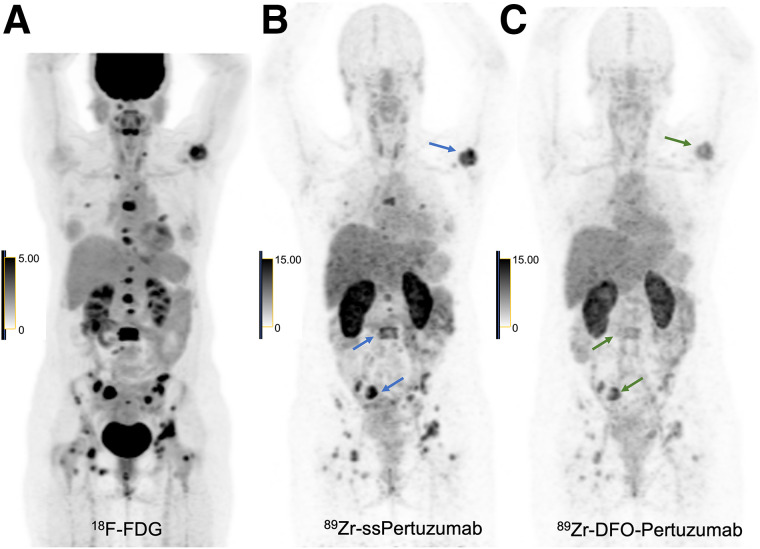
46-y-old woman with HER2-positive mBC and known osseous metastases. (A) ^18^F-FDG maximum-intensity projection PET image demonstrating numerous avid osseous metastases. (B and C) Maximum-intensity projection PET images of ^89^Zr-ss-pertuzumab (B) and ^89^Zr-DFO-pertuzumab (C) at 5 d after injection showing uptake within osseous metastases; however, ^89^Zr-ss-pertuzumab detected more lesions and had higher lesion conspicuity and intensity of tracer uptake. The 3 hottest lesions with ^89^Zr-ss-pertuzumab and ^89^Zr-DFO-pertuzumab are denoted with arrows. ^89^Zr-ss-pertuzumab PET/CT was performed 1 mo before ^89^Zr-DFO-pertuzumab PET/CT.

This patient also underwent both ^89^Zr-ss-pertuzumab PET/CT and ^89^Zr-DFO-pertuzumab PET/CT 1 mo apart during a treatment holiday, with ^89^Zr-ss-pertuzumab PET/CT performed first. Representative images (Fig. 4) show markedly improved lesion detection, conspicuity, and intensity of tracer uptake by numerous osseous lesions with ^89^Zr-ss-pertuzumab compared with ^89^Zr-DFO-pertuzumab. For example, the total number of tracer-avid osseous lesions visualized on ^89^Zr-ss-pertuzumab PET/CT was 55, versus 38 on ^89^Zr-DFO-pertuzumab PET/CT. For ^89^Zr-ss-pertuzumab versus ^89^Zr-DFO-pertuzumab, the SUV_max_ of the 3 hottest lesions was 22.3 versus 12.2, respectively, in the right sacrum, 15.5 versus 6.9, respectively, in L3, and 19.7 versus 8.9, respectively, in the left humerus.

Patient 3 had an ER-positive, PR-positive, HER2-positive (IHC score, 3+) right-breast mixed ductal and lobular invasive carcinoma diagnosed in 2021, with de novo metastatic disease to the right axillary nodes, liver, and bones. A left iliac bone biopsy was ER-positive, PR-positive, and HER2-positve (IHC score equivocal because of crush artifact, but *ERBB2* amplification on next-generation sequencing). She had just completed her fourth cycle of paclitaxel, trastuzumab, and pertuzumab before ^89^Zr-ss-pertuzumab PET/CT. No tracer-avid lesions were visualized, and the lack of ^89^Zr-ss-pertuzumab uptake in known metastatic lesions was thought to be due to treatment response (as opposed to lack of HER2 expression), as an ^18^F-FDG PET/CT shortly afterward showed complete response with no ^18^F-FDG–avid disease.

Patient 4 had an ER-positive, PR-negative, HER2-positive (IHC score, 2+; FISH ratio, 2.6) left-breast IDC diagnosed in 2019, with metastatic disease to the bones. An L2 vertebral metastasis was biopsied as HER2-negative (IHC score, 1+). The patient was started on paclitaxel, trastuzumab, and pertuzumab, and the osseous metastasis progressed. Given the HER2 discordance, a repeat HER2 FISH was performed on the primary breast tumor and was HER2-negative (FISH ratio, 1.87). He underwent multiple lines of therapy and had recently progressed on trastuzumab and pertuzumab before being referred for ^89^Zr-ss-pertuzumab PET/CT to assess the current HER2 status of his disease and decide whether to continue HER2-targeted therapy or switch to chemotherapy. At enrollment, he had ^18^F-FDG–avid diffuse osseous metastases and multiple hepatic metastases. Of the diffuse osseous disease, only a few lesions demonstrated only mild ^89^Zr-ss-pertuzumab avidity, and there were no tracer-avid hepatic lesions. Since only a small percentage was HER2 PET–positive (concordance, 7.4%), he was switched to chemotherapy with doxorubicin instead of HER2-targeted therapy.

Patient 5 had an ER-negative, PR-negative, HER2-positive (IHC score, 3+) right-breast IDC diagnosed in 2011 and underwent neoadjuvant chemotherapy and mastectomy followed by 1 y of adjuvant trastuzumab. In 2017, she was found to have pulmonary nodules, one of which was biopsied and found to be HER2-positive (IHC score, 3+). She received multiple lines of HER2-targeted therapy including paclitaxel, trastuzumab, and pertuzumab; trastuzumab emtansine; and capecitabine and neratinib but subsequently developed brain and bone metastases. She was started on T-DXd and had a partial response but was lost to follow-up for several months. She was restarted on T-DXd but developed progression of ^18^F-FDG–avid lung and bone metastases. The patient was referred for ^89^Zr-ss-pertuzumab PET/CT to assess her current HER2 status and help decide between further HER2-targeted therapy or chemotherapy. HER2 PET heterogeneity was observed with ^89^Zr-ss-pertuzumab avidity in a solitary sternal lesion on day 7 (SUV_max_, 12.7), whereas the remaining bone and lung lesions were not tracer-avid. Since at least 1 lesion was tracer-avid, she was switched to another line of HER2-targeted therapy with trastuzumab, capecitabine, and tucatinib.

Patient 6 had an ER-positive, PR-positive, HER2-positive (IHC score, 2+; FISH ratio, 2.1) left-breast IDC diagnosed in 2016 and underwent neoadjuvant chemotherapy, lumpectomy, and radiation followed by adjuvant letrozole. She was subsequently diagnosed with metastases to the lung, neck and thoracic nodes, and sternum. A left neck node was biopsied and found to be HER2-positive (IHC score, 2+; FISH ratio, 2.34). She was treated with several lines of HER2-targeted therapy, with progression of the osseous metastases and a subcarinal node. The patient was referred for ^89^Zr-ss-pertuzumab PET/CT to assess her current HER2 status and help decide between further HER2-targeted therapy or chemotherapy. HER2 PET heterogeneity was observed, with gradually increasing ^89^Zr-ss-pertuzumab avidity in all bone lesions on days 1, 3, and 6 after injection (SUV_max_ of the most avid sternal lesions, 11.7, 18.5, and 34.4) but no uptake in a mildly ^18^F-FDG–avid subcarinal nodal metastasis. Since most lesions demonstrated ^89^Zr-ss-pertuzumab avidity, she was switched to another line of HER2-targeted therapy with trastuzumab, capecitabine, and tucatinib.

## DISCUSSION

In this trial, we demonstrated the safety and investigated the biodistribution and dosimetry of ^89^Zr-ss-pertuzumab. We also showed that ^89^Zr-ss-pertuzumab successfully targets and visualizes metastatic lesions in patients with HER2-positive mBC. To our knowledge, this is first successful clinical translation of a site-specifically radiolabeled antibody.

Similar to two other HER2-targeted immuno-PET agents, ^89^Zr-DFO-trastuzumab and ^89^Zr-DFO-pertuzumab, the optimal imaging time point for ^89^Zr-ss-pertuzumab was 5–8 d. Tumor uptake increased over time, and concomitant decreases were seen in the blood pool and liver background. The mean effective dose of ^89^Zr-ss-pertuzumab (0.54 mSv/MBq) was equal to that of ^89^Zr-DFO-pertuzumab (0.54 mSv/MBq) (10) and comparable to that of ^89^Zr-DFO-trastuzumab (0.48 mSv/MBq) (9). The biodistribution and normal-tissue dosimetry of ^89^Zr-ss-pertuzumab were also similar; however, ^89^Zr-ss-pertuzumab had the highest uptake in the kidneys, whereas the other two had the highest uptake in the liver. Otherwise, the dosimetry profile was relatively similar among the 3 tracers. One patient (patient 2) underwent PET/CT with both ^89^Zr-ss-pertuzumab and ^89^Zr-DFO-pertuzumab 1 mo apart during a treatment break, allowing for a preliminary comparison between the site-specifically and stochastically lysine-labeled radioimmunoconjugates. ^89^Zr-ss-pertuzumab showed tumor lesion detection, conspicuity, and uptake superior to those of ^89^Zr-DFO-pertuzumab in this patient, in keeping with the superior imaging properties observed in preclinical models (23). Compared with trastuzumab, radiolabeling of pertuzumab has advantages since pertuzumab binds to a different site (extracellular domain II) on the HER2 receptor from trastuzumab (domain IV) (24), preventing the radioimmunoconjugate from interfering with drug binding in breast cancer patients treated with trastuzumab-based agents. However, radiolabeled pertuzumab may be limited in potential applications such as predicting response to trastuzumab-based therapy, including the newer HER2-targeted antibody–drug conjugates.

In our study, ^89^Zr-ss-pertuzumab PET/CT was used to assess the current HER2 status of metastatic lesions to guide biopsy and assist in deciding the next line of treatment. Although CT and ^18^F-FDG PET/CT are excellent in detecting tumor lesions in mBC (25), these imaging modalities provide information only on the presence or absence, size, and viability of tumor lesions, with no information on their current receptor status. Thus, a potential application of ^89^Zr-ss-pertuzumab PET/CT is to select a lesion to biopsy in patients with more than 1 metastatic lesion. Lesion selection is critically important, as one of the primary goals of tissue sampling is to maximize the chances of identifying HER2-positive disease so a patient can receive HER2-targeted therapy.

In the first 2 patients, ^89^Zr-ss-pertuzumab PET/CT helped select lesions to biopsy, as patient 1 had more than 5 hepatic lesions and patient 2 had more than 20 osseous lesions. In both patients, lesion selection was based on ^89^Zr-ss-pertuzumab avidity and biopsy feasibility. Surprisingly, both lesions were found to be HER2-negative on pathology despite clear and intense tracer avidity (SUV_max_ range, 22.3–30.0). One possible explanation of this phenomenon is that ^89^Zr-ss-pertuzumab uptake does not necessarily align with the historical binary definition of HER2 status per the guidelines of the American Society of Clinical Oncology and the College of American Pathologists. Rather, ^89^Zr-ss-pertuzumab may be able to detect a wide array of HER2 expression levels and thus may visualize both HER2-positive tumors and HER2-low tumors (IHC score, 1+, or 2+ and FISH negative). This hypothesis is in line with the recent findings of the DESTINY-Breast04 trial, which showed for the first time that HER2-targeted therapy with T-DXd had efficacy in patients with HER2-low mBC (26), thereby establishing HER2-low patients as a new and distinct population (26,27). This detection of HER2-low tumors may also in part explain the false positives for HER2-positive malignancy in our prior HER2 PET studies (7,8,11).

Another potential application for ^89^Zr-ss-pertuzumab PET/CT is real-time assessment of intertumoral HER2 expression heterogeneity throughout all lesions to assist in deciding whether to continue HER2-targeted therapy or change to chemotherapy at disease progression. In our study, 3 patients—patients 4, 5, and 6—underwent ^89^Zr-ss-pertuzumab PET for this reason. Patient 4 had positive ^89^Zr-ss-pertuzumab PET findings, but only a small percentage of his diffuse disease was tracer-avid and thus he was switched to chemotherapy. Patients 5 and 6 had positive ^89^Zr-ss-pertuzumab PET findings with at least 1 tracer-avid lesion and were thus switched to another line of HER2-targeted therapy. Interestingly, whereas both patients had positive HER2 PET findings, different degrees of HER2 intertumoral heterogeneity were observed on a lesion-by-lesion basis: patient 5 had only 1 ^89^Zr-ss-pertuzumab–avid lesion of 5 total (20% concordance), whereas patient 6 had 4 ^89^Zr-ss-pertuzumab–avid lesions of 5 total (80% concordance).

It remains unclear at this point whether patients with more homogeneous and uniform HER2 expression will respond better to HER2-targeted therapy than patients with more heterogeneous and discordant HER2 expression. Mechanistically, however, this hypothesis is certainly plausible, especially in light of the growing evidence supporting the theranostic utility of other PET tracers such as ^68^Ga-DOTATATE (28) and ^68^Ga-PSMA-617 (29). Along these lines, the ZEPHIR trial demonstrated that ^89^Zr-DFO-trastuzumab PET/CT predicted treatment response to trastuzumab emtansine in mBC patients with a positive predictive value of 72% and negative predictive value of 88%. In addition, 29% of patients in this study had negative HER2 PET findings and 46% of patients had intertumoral heterogeneity, further highlighting how single-site biopsies may underestimate underlying HER2 heterogeneity. In this study, ^89^Zr-DFO-trastuzumab PET/CT results were classified as positive if more than 50% of the tumor load was tracer-avid and as negative if otherwise (4). However, no clear method has yet been established for HER2 PET interpretation or defining HER2 PET heterogeneity.

^89^Zr-ss-pertuzumab is a promising HER2 immuno-PET agent. Further exploration is necessary, and we plan to continue this work through an ongoing pancancer trial investigating ^89^Zr-ss-pertuzumab PET/CT in patients with different HER2-positive primary cancers. Other areas of investigation include whether ^89^Zr-ss-pertuzumab can detect brain metastases, since the rate of brain metastasis is higher in patients with HER2-positive mBC than in those with HER2-negative disease (30). Finally, the landmark approval of T-DXd, a HER2-targeted antibody–drug conjugate, for patients with HER2-low mBC may portend yet another clinical application for ^89^Zr-ss-pertuzumab PET, potentially as a predictive biomarker for this novel antibody–drug conjugate.

## CONCLUSION

This study demonstrated the safety, dosimetry, and tumor targeting of ^89^Zr-ss-pertuzumab PET and represents the first translation of a site-specifically radiolabeled antibody to the clinic. Potential clinical applications include assessment of current HER2 status and the heterogeneity of metastatic lesions throughout the body to guide biopsy and treatment decisions.

## DISCLOSURE

Funding was received from National Institutes of Health (NIH) grant R01 CA204167 (to Brian Agnew, Gary Ulaner, Jason Lewis, and Brian Zeglis) and from the Memorial Sloan Kettering Cancer Center Radiochemistry and Molecular Imaging Probe Core (NIH grant P30 CA08748) for additional support. Jason Lewis is supported in part by R35 CA232130. Shanu Modi serves as a scientific advisor/consultant for Genentech, Daiichi Sankyo, AstraZeneca, Seagen, Gilead, Macrogenics, Zymeworks, and Novartis; receives honoraria from Daiichi Sankyo, AstraZeneca, and Seagen; and has institutional grants for research from Genentech, Daiichi Sankyo, AstraZeneca, and Seagen. No other potential conflict of interest relevant to this article was reported.

## Notes

KEY POINTS **QUESTION:** What are the safety, radiation dosimetry, and potential imaging characteristics of site-specifically radiolabeled ^89^Zr-pertuzumab, and how does it compare with prior HER2-targeted immuno-PET probes? **PERTINENT FINDINGS:** In a first-in-humans clinical trial on 6 patients with HER2-positive mBC, ^89^Zr-ss-pertuzumab PET/CT was safe and demonstrated biodistribution and dosimetry profiles similar to those of ^89^Zr-DFO-pertuzumab and ^89^Zr-DFO-trastuzumab. ^89^Zr-ss-pertuzumab PET/CT was able to detect known malignant lesions, indicating that they are HER2-positive, and may detect more lesions than ^89^Zr-DFO-pertuzumab. **IMPLICATIONS FOR PATIENT CARE:** HER2 PET with ^89^Zr-ss-pertuzumab could enable real-time evaluation of HER2 status to guide biopsy and treatment decisions.
